# Evaluation of Dengue NS1 Antigen Rapid Tests and ELISA Kits Using Clinical Samples

**DOI:** 10.1371/journal.pone.0113411

**Published:** 2014-11-20

**Authors:** Subhamoy Pal, Allison L. Dauner, Indrani Mitra, Brett M. Forshey, Paquita Garcia, Amy C. Morrison, Eric S. Halsey, Tadeusz J. Kochel, Shuenn-Jue L. Wu

**Affiliations:** 1 Naval Medical Research Center, Silver Spring, Maryland 20910-7500, United States of America; 2 U.S. Naval Medical Research Unit, No. 6, Lima, Peru; 3 Peruvian Ministry of Health, Instituto Nacional de Salud, Lima, Peru; 4 University of California Davis, Davis, CA, 95616, United States of America; Naval Research Laboratory, United States of America

## Abstract

**Background:**

Early diagnosis of dengue virus (DENV) infection can improve clinical outcomes by ensuring close follow-up, initiating appropriate supportive therapies and raising awareness to the potential of hemorrhage or shock. Non-structural glycoprotein-1 (NS1) has proven to be a useful biomarker for early diagnosis of dengue. A number of rapid diagnostic tests (RDTs) and enzyme-linked immunosorbent assays (ELISAs) targeting NS1 antigen (Ag) are now commercially available. Here we evaluated these tests using a well-characterized panel of clinical samples to determine their effectiveness for early diagnosis.

**Methodology/Principal Findings:**

Retrospective samples from South America were used to evaluate the following tests: (i) “Dengue NS1 Ag STRIP” and (ii) “Platelia Dengue NS1 Ag ELISA” (Bio-Rad, France), (iii) “Dengue NS1 Detect Rapid Test (1^st^ Generation)” and (iv) “DENV Detect NS1 ELISA” (InBios International, United States), (v) “Panbio Dengue Early Rapid (1^st^ generation)” (vi) “Panbio Dengue Early ELISA (2^nd^ generation)” and (vii) “SD Bioline Dengue NS1 Ag Rapid Test” (Alere, United States). Overall, the sensitivity of the RDTs ranged from 71.9%–79.1% while the sensitivity of the ELISAs varied between 85.6–95.9%, using virus isolation as the reference method. Most tests had lower sensitivity for DENV-4 relative to the other three serotypes, were less sensitive in detecting secondary infections, and appeared to be most sensitive on Day 3–4 post symptom onset. The specificity of all evaluated tests ranged from 95%–100%.

**Conclusions:**

ELISAs had greater overall sensitivity than RDTs. In conjunction with other parameters, the performance data can help determine which dengue diagnostics should be used during the first few days of illness, when the patients are most likely to present to a clinic seeking care.

## Introduction

Up to 390 million dengue cases are thought to occur each year, and approximately 2.5 billion people are at risk for infection worldwide, with no vaccine or antiviral approved to reduce disease burden [1], [2]. Accurate and affordable diagnostic tests are a crucial component of combating this debilitating mosquito-borne infection. Such assays would permit early diagnosis of dengue and thus improve clinical management of patients. Dengue is caused by any of four serotypes of dengue virus (DENV-1, 2, 3 and 4), a single-stranded, positive sense enveloped RNA virus that belongs to the genus *Flavivirus* [3], [4]. During outbreaks, the number of people reporting to clinics with severe disease can overwhelm the public health systems of many urban centers. Differential diagnosis based on symptoms is challenging due to dengue's non-specific symptoms such as fever, aches and fatigue that often overlap with other endemic infections. Dengue-associated mortality can be reduced from 20–30% in severe cases to less than 1% with appropriate fluid replacement and supportive care, which is greatly facilitated by early diagnosis [5]–[7]. A positive laboratory test often alerts physicians to closely monitor platelet levels and other disease specific warning symptoms associated with severe disease. From a public health perspective, identification of dengue can geographically focus countermeasures such as targeted vector control.

While early and appropriate management of dengue is correlated with better outcome [8], no single laboratory test can be used to accurately diagnose disease over the course of illness. Traditional laboratory techniques for dengue diagnosis include detection of RNA using reverse transcription polymerase chain reaction (RT-PCR) or viral isolation followed by indirect immunofluorescence assay (IFA); both methods are effective during the first five days of illness and tend to decrease in sensitivity as viremia wanes over time [9]–[11]. However, RT-PCR requires specialized reagents and trained personnel, while virus isolation can take days or weeks to complete. The most widely used method for diagnosing dengue is an enzyme-linked immunosorbent assay (ELISA) which measures anti-DENV IgM or IgG antibodies in patient serum. These antibodies are not reliably detectable until 3–4 days post symptom onset (PSO) [12] and requires the collection of a second blood sample 14–21 days after the first visit for a definitive diagnosis. Serological diagnosis does not therefore inform immediate treatment decisions during acute illness. Since dengue patients often present within 3 days PSO (unpublished observation), false negative results with antibody-based assays remain a concern. Numerous successful molecular assays have been developed that detect DENV nucleic acid within the acute phase of illness [13]–[17]; however, these RT-PCR or isothermal molecular detection systems have yet to transition from the laboratory to a point-of-care format.

There are now a number of assays in development or on the market for diagnosing dengue during the acute stage of infection [18], [19]. In 2000, the first ELISA capable of detecting DENV non-structural protein-1 (NS1) was developed. NS1 is found in both membrane and soluble forms and is highly conserved [20]. A soluble hexameric form of NS1 is released during DENV infection and accumulates in high concentrations (up to 50 µg/ml) in human serum [21], [22]. Importantly, NS1 is detectable early during the acute phase (Day 0 to 6 PSO) of both primary and secondary DENV infections [21], [23]. Together, the magnitude and timing of NS1 levels in human clinical specimens makes it an attractive target for diagnostic assay development [20], [22], [24]. Recent work has demonstrated that an NS1 antigen-based assay used in conjunction with a serological diagnostic marker (e.g., anti-DENV IgM) can enhance the sensitivity and specificity of dengue diagnosis through all stages of the disease [25]–[28]. Furthermore, quantitative detection of NS1 may help predict the risk associated with DENV infection, as high NS1 levels have been found to correlate with dengue hemorrhagic fever (DHF) [21], [23], [29], [30]. Recently, NS1 tests have also been reported to be effective for detecting DENV in vector populations [31], [32]. These tests can therefore improve both clinical management and vector surveillance.

The objective of this study was to compare seven commercially available DENV NS1 tests, four RDTs and three ELISAs, utilizing serum samples from confirmed DENV-infected and uninfected febrile patients collected in an endemic setting in Peru. While other groups have evaluated some of these assays in the past [26], [27], [33]–[37], this study also includes new dengue NS1 tests developed by InBios, Inc. This is also the first assessment of dengue NS1 products utilizing DENV strains circulating in Peru. Overall, these results add to the growing body of literature about NS1 test performance and may aid public health decision-makers in selecting tests for specific applications.

## Methods

### Human Use Statement

The procedures applied in this study were done in accordance with the ethical standards of the Naval Medical Research Center (NMRC; Silver Spring, MD) Institutional Review Board and with the Helsinki Declaration of 1975, as revised in 1983. Study protocols were approved by the NMRC and Naval Medical Research Unit No. 6 (NAMRU-6; Lima, Peru) Institutional Review Boards (NMRCD.2000.0006 and NMRCD.2001.0002) in compliance with all applicable federal regulations governing the protection of human subjects. Study protocols were also reviewed by public health authorities in Peru (Instituto Nacional de Salud). Written consent was obtained from subjects 18 years of age and older. For younger participants written consent was obtained from a parent or legal guardian, and written assent was obtained from the participant.

### Clinical Samples

Evaluation of the NS1 assays was conducted at NMRC using serum samples from a Surveillance and Etiology of Acute Febrile Illnesses in Peru (Study protocol NMRCD.2000.0006) at regional sites in Piura, Tumbes, Madre de Dios, and Iquitos. Surveillance and Etiology of Acute Febrile Illnesses in Ecuador and Honduras was performed under study protocol NMRCD.2001.0002, with samples from Honduras collected at the Instituto Hondureño de Seguridad Social, Tegucigalpa. Patients with an acute febrile illness were enrolled when reporting fever (≥38°C oral, tympanic, or rectal; ≥37.5°C axillary) for five days or less, accompanied with headache, muscle, ocular and/or joint pain, to public, military or private health facilities around regional sites. Symptoms and demographic information were collected.

### DENV Reference Testing

#### Virus Isolation

Virus isolation was attempted for all acute samples, and DENV was identified using serotype-specific IFAs. Briefly, African green monkey Vero (37°C) and *Aedes albopictus* mosquito C6/36 (28°C) cell cultures were each inoculated with diluted serum. Upon observation of cytopathic effect (CPE), or ten days post-inoculation if no CPE was observed, cells were removed from the flasks and prepared for microscopic examination by standard indirect IFA. This was followed by the addition of fluorescein-conjugated goat anti-mouse IgG. DENV serotypes were identified using serotype-specific monoclonal antibodies (DENV-1: 15F3, DENV-2: 3H5; DENV-3: 5D4; DENV-4: 1H10).

#### Serology

DENV IgM and IgG titers were determined by ELISA, as previously described [38], [39]. Viral antigens for the ELISAs were produced at the NAMRU-6-Lima laboratory from pooled supernatants of infected Vero cell cultures using DENV-1 West Pac 74, DENV-2 S16803, DENV-3 CH53489, and DENV-4 TVP-360. Prior to homogenization, antigen preparations were inactivated using 3 mM binary ethylenimine. Any acute sample for which IgG levels were below the cut-off for positivity or for which IgM/IgG ratio was>0.5 was defined as a primary infection. High levels of IgG in acute samples defined secondary infections. As part of ongoing surveillance protocols, the serum samples were also routinely tested by IFA and IgM capture ELISA for evidence of recent infection by a panel of zoonotic and vector-borne pathogens, including alphaviruses, orthobunyaviruses, and arenaviruses [40]. Non-DENV pathogens tested for included St. Louis encephalitis, yellow fever, West Nile, Venezuelan equine encephalitis, Eastern equine encephalitis, mayaro, oropouche, Q-fever, and typhi and rickettsii group of rickettsias. Previously identified reactive sera were used as positive controls, and DENV-uninfected human serum was used as a negative control. Samples exceeding the reference cut-off value, calculated as the mean of seven antibody-negative samples (normal human serum) plus three standard deviations, were considered antibody positive. Positive samples were subsequently re-tested at four-fold serial dilutions (1∶100, 1∶400, 1∶1600, and 1∶6400).

### Commercial tests for DENV NS1 antigen detection

Seven commercially available dengue NS1 kits were evaluated, including four RDTs and three in ELISA format. These kits were: “Dengue NS1 Ag STRIP” and “Platelia Dengue NS1 Ag ELISA” (Bio-Rad, France), “Dengue NS1 Detect Rapid Test (1^st^ generation)” and “DENV Detect NS1 ELISA” (InBios International, United States), “Panbio Dengue Early Rapid”, “Panbio Dengue Early ELISA (2^nd^ generation)” and “SD Bioline Dengue NS1 Ag Rapid Test” (Alere, United States). All kits are available outside the United States for research use or commercial purchase. Assays were performed according to the manufacturers' instructions. All kits were tested using serum specimens. All RDTs had a control line and a test line. The appearance of the test and control lines after a specified migration time (15–30 minutes) indicated a positive result. The appearance of the control line alone indicated a negative result. The technicians carrying out the evaluation of the test articles were blind to the DENV-infection status of the panel of serum samples. For each RDT involving the interpretation of the presence of a line, two people read the results independently and concurred on a given call. The ELISAs were also performed per the manufacturers' protocols, with absorbance measured at 450/620 nm within 30 minutes of the addition of stop buffer.

### Statistical Methods

Sensitivity, specificity, and agreement were calculated with reference to the “gold standard” reference methodology using widely accepted definitions [41]. Confidence intervals for sensitivity and specificity were calculated using the Exact Binomial method [42]. Z-ratio's for the significance of the difference between two independent ratios were performed to test whether a given sensitivity (or specificity) was statistically different from another, and a p-value <0.05 (two-tailed) was considered to be statistically significant [43].

## Results

### Characteristics of the study population and test devices

**Figure 1:**
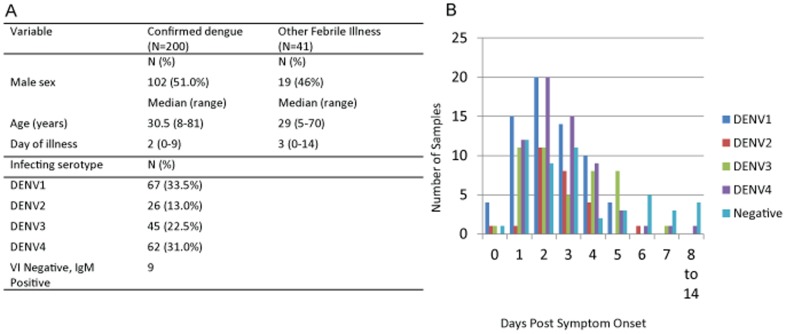
Characteristics of clinical samples used in this study. A. Sex, age, day of illness, and infecting serotype for subjects. B. A plot of the number of samples for a given infecting serotype of DENV graphed over day post symptom onset.

Initially, 250 acute serum samples from febrile individuals were obtained for this study (Figure 1A). Serum samples were characterized for DENV infection using traditional laboratory techniques that have been described previously [40]. We utilized 200 samples categorized as DENV-positive based on virus isolation and 41 samples categorized as DENV-negative based on testing by virus isolation and serology. Nine samples initially categorized as DENV-negative based on absence of virus isolation were later found to be positive for DENV IgM. These samples were excluded from further analysis, as ELISA results alone were not considered to be specific enough to confirm acute DENV infection. None of the isolation-negative samples used for this study had IgM titers against any other flavivirus infections tested (see methods for testing panel), with the exception of one sample that had IgM titers suggestive of prior St. Louis encephalitis virus exposure. Due to volume restrictions, not all samples were tested on all products.

Confirmed dengue cases and other febrile illnesses (OFI) were both evenly distributed between male and female; 51% male and 54% male respectively (Figure 1). The age distribution was unimodal, with a median age of 30 years, and an age range from five to 81 years. The median day of sample collection PSO was Day 2 (range 0–14). Greater than 80% of our samples were collected between Days 1–4 PSO (Figure 1B). DENV-infected samples included all four DENV serotypes (Figure 1).

A comparison of several functional attributes of each product is presented in Table 1. The indications for use ranged from serum only (Panbio, InBios) to EDTA-treated whole blood, serum, and plasma (SD). Only the serum claim was evaluated in this study. As expected, the NS1 ELISA kits required several additional steps when compared to RDTs and approximately 2–3 hours of assay time. RDTs required 15–30 minutes. The volume of sample required for the SD RDT (three drops corresponding to about 105 µl) was higher than other RDTs which required 50 µl. Only the SD and InBios RDTs can be stored at room temperature while the others need to be refrigerated at 2–8°C.

**Table 1:** Characteristics of dengue NS1 diagnostics.

Assay type	Rapid diagnostic tests				ELISA format assays		
Manufacturer	BioRad	InBios	Panbio	SD	BioRad	InBios	Panbio
Blood matrices	Plasma, sera	Sera	Sera	EDTA-treated blood, plasma, sera	Plasma, sera	Sera	Sera
Assay time (Minutes)	15–30	30	15	15–20	140	111	160
Volume necessary	50 µL	50 µL	50 µL	105 µL	50 µL	50 µL	75 µL
Format	Dipstick	Dipstick	Dipstick	Cassette	96-well	96-well	96-well
Extra materials required	Tubes, pipette	Pipette, tubes	Pipette	No	Pipette, incubator, plate reader	Pipette, incubator, plate reader	Pipette, incubator, plate reader
Storage	2–8°C	Room Temp.	2–8°C	Room Temp.	2–8°C	2–8°C	2–8°C

Matrix, assay time, required volume, required additional equipment and storage temperature for each diagnostic test.

### Test device performance

Among RDTs, Bio-Rad demonstrated the highest overall sensitivity of 79.1% (95% C.I. 71.8–85.2%), followed by InBios (76.5%; 64.6–85.9%), SD (72.4%; 64.5–79.3%) and Panbio (71.9%; 64.1–78.9%; Table 2). The specificity for each test was as follows: Bio-Rad (100%; 95% C.I. 91.1–100.0%), InBios (97.3%; 86.2–99.9%), SD (100%; 91.1–100%) and Panbio (95.0%; 83.1–99.4%). The loss in overall sensitivity was due to the very low sensitivity of all tests to DENV-4 (Table 2), with only 58.1% (Bio-Rad), 53.5% (SD), 44.2% (Panbio) and 42.1% (InBios) sensitivity. The sensitivity was highest to DENV-1 for all four RDTs, ranging from 91.4 to 95.2%, while sensitivity to DENV-2 and DENV-3 ranged from 69.2 to 87.5%.

**Table 2:** Device sensitivity and specificity: Numbers of samples tested for RDTs and ELISAs, to show serotype-specific as well as overall sensitivity and specificity.

Assay type	Rapid diagnostic test				ELISA format assays		
Manufacturer	BioRad	InBios	Panbio	SD	BioRad	InBios	Panbio
DENV1	95.2% (40/42)	91.4% (32/35)	92.9% (39/42)	92.9% (39/42)	100.0% (45/45)	93.8% (30/32)	96.4% (53/55)
DENV2	76.9% (20/26)	83.3% (5/6)	80.8% (21/26)	69.2% (18/26)	80.0% (12/15)	100.0% (6/6)	94.1% (16/17)
DENV3	85.7% (36/42)	87.5% (7/8)	73.8% (31/42)	73.2% (30/41)	96.3% (26/27)	100.0% (5/5)	89.7% (26/29)
DENV4	58.1% (25/43)	42.1% (8/19)	44.2% (19/43)	53.5% (23/43)	75.0% (27/36)	100.0% (6/6)	65.9% (29/44)
Overall sensitivity	79.1% (121/153)	76.5% (52/68)	71.9% (110/153)	72.4% (110/152)	89.4% (110/123)	95.9% (47/49)	85.5% (124/145)
Overall specificity	100.0% (0/40)	97.4% (1/38)	95% (2/40)	100.0% (0/40)	97.4% (1/38)	100.0% (0/36)	95.0% (2/40)

We found the following overall sensitivity for each ELISA: InBios 95.9% (95% C.I. 86.0–99.5%), Bio-Rad 89.4% (82.6–94.3%) and Panbio 85.6% (78.9–90.9%; Table 2). Sensitivity exceeded 90% for DENV-1 for all three ELISA kits. DENV-4 sensitivity varied the most, ranging from 100% (InBios) to 75.0% (Bio-Rad) and 66.7% (Panbio). The overall sensitivity for ELISA kits was higher than that for the RDTs. Specifically, the Bio-Rad ELISA was significantly more sensitive than all RDT's (p = 0.02, p = 0.02, p<0.001, and p<0.001 compared to the Bio-Rad, InBios, Panbio, and SD RDTs, respectively), while the Panbio ELISA was significantly more sensitive than the Panbio and SD RDT's (p = 0.004 and p = 0.006, respectively). The corresponding overall specificities for each ELISA kit were: InBios 100.0% (95% C.I. 90.3–100%), Bio-Rad 97.3% (86.2–99.9%), and Panbio 95.0% (83.1–99.4%), which were similar to the RDTs.

Very few false positive results were obtained from OFI samples for any of the kits; the resulting specificity for all test articles was determined to be between 95–100%. Only three of the OFI samples were reactive to one or more test articles. No specific etiology was identified for these samples. One sample collected on day 1 PSO was reactive to both the Panbio RDT and ELISA, and one sample from day 5 PSO was positive using both the Panbio and Bio-Rad ELISA; the remaining sample, from day 7 PSO, was reactive to the Panbio RDT but was not reactive to any other test article.

**Figure 2:**
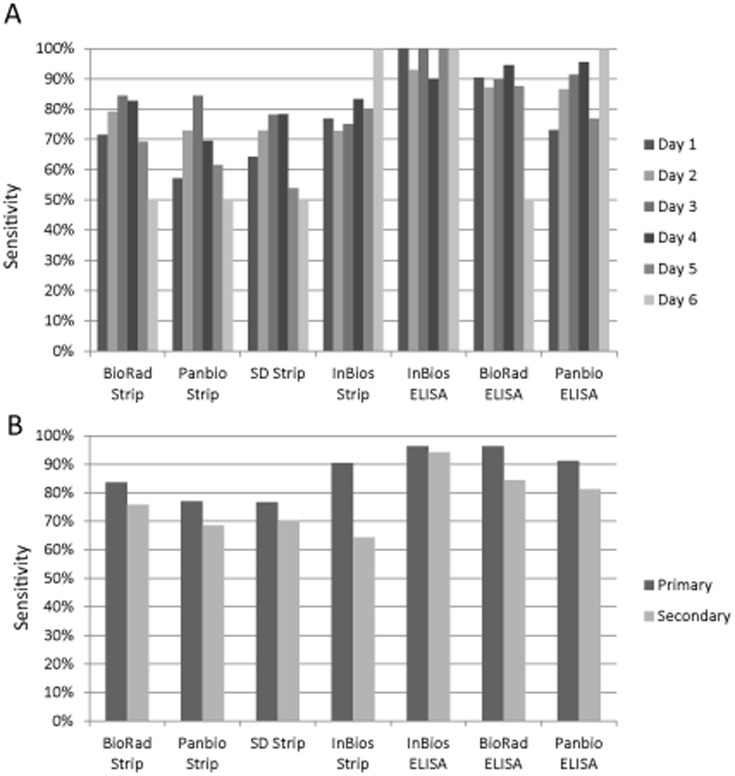
Device performance stratified by day post symptom onset and infection status. The diagnostic tests are shown on the X-axis, while the sensitivity of the device is shown in the Y-axis. Color of the bar denotes day PSO (A) or primary/secondary infection status (B).

The highest sensitivity of the NS1 assays was generally found between days 2–4 PSO. The Bio-Rad and Panbio RDTs demonstrated peak sensitivity on day 3 PSO (84.4%), while SD and InBios RDTs peaked on day 4 (78.3% and 83.3%, respectively; Figure 2A). The Bio-Rad and Panbio ELISAs also displayed peak sensitivity on day 4 PSO. Most RDTs generally showed decreasing sensitivity for every day removed from the peak. Among DENV positive samples, 81 samples were characterized as a primary infection and 90 samples were characterized as secondary infection (Figure 2B). On average, all tests were 10.5% (range: 2% for InBios ELISA to 26% for InBios RDT) more sensitive for detecting primary dengue infections compared with secondary infections, as has been reported by others [34], [44]. Overall, the ELISAs displayed better sensitivity than RDTs at all time points.

## Discussion

RDTs that can be performed near the patient's point-of-care are being adopted worldwide for their utility in initial diagnosis. Simple assays capable of providing an answer within 15–30 minutes of sampling are highly desirable, especially in resource-limited settings. The performance of the RDTs will need to be weighed in context with other attributes that may be important to the end user (Table 1) including local market price, sample matrix that can be used, volume of sample necessary, storage temperature and shelf life. We found that ELISA kits had superior sensitivity when compared to RDTs. Because of their superior performance, ELISAs would be the recommended diagnostic choice when laboratories with trained personnel and equipment are available.

Depending on the prevalence of dengue and other febrile diseases, the positive and negative predictive values of the devices tested will vary. However, given the high specificity observed for both RDTs and ELISAs, the positive predictive value (PPV) of these devices is expected to be greater than 85% in most endemic countries, where dengue accounts for over 30% of febrile disease (PPV ranging from 86% for the Panbio RDT to 100% for the Bio-Rad RDT, SD RDT, and InBios ELISA). Thus individuals testing positive are unlikely to require further confirmatory testing. We found few RDTs or ELISAs reacting to the OFI samples, but an explicit cross-reactivity panel was not performed; false positives as a result of a cross-reactive antigen can adversely affect the PPV. RDTs had lower sensitivity than ELISAs, consequently the negative predictive value of an NS1 ELISA is likely to be superior to that of RDTs. Individuals testing negative on an RDT but still presenting with high clinical suspicion of dengue could be re-tested using laboratory assays, which may include a combination of NS1 ELISA, RT-PCR, and MAC-ELISA. Even so, RDTs can have considerable utility by significantly reducing the amount of confirmatory testing required.

Three factors appeared to correlate with the likelihood that a given sample will produce a false negative result. The first factor was the day PSO. Most tests achieved maximum sensitivity on days 2–4 PSO. This is likely correlated with temporal changes in NS1 antigen levels in patient sera [33]. Previous studies have shown that NS1 antigenemia fluctuates throughout disease with detectable levels occurring with the start of illness. NS1 levels have been shown to peak around day 4–5 PSO during primary infections, but wane earlier in secondary infections [23]. The second factor was the infection status: we found overall lower sensitivity in secondary infections for all test articles. This phenomenon has been previously observed and may be due to antibodies against DENV NS1 in the patient sample forming antigen-antibody complexes, thereby reducing access to the target epitopes for the test articles [34], [45]. The third factor potentially contributing to false negative results was the infecting DENV serotype. This factor was most pronounced in the RDTs, where DENV-4 sensitivity averaged only 50%. Other groups have reported different sensitivities for NS1 diagnostic tests for DENV-2 and DENV-4 [36], [46], using both clinical samples and tissue culture-derived virus. A number of reasons may exist for this: (i) large antigenic distance between circulating DENV-4 strains (from Peru in this case) and the antibodies used in the commercial assays leading to poor binding, or (ii) lower overall viremia and NS1 antigen levels in DENV-4 infections making it a less abundant target [34], [47].

Certain limitations of this study relate to the types of samples used. The study was performed using retrospective samples, and the study results would have been even more directly applicable had it been performed prospectively during the course of routine dengue surveillance activities. Using a panel of well-characterized samples eliminates borderline or weak positive samples which would likely be included if the evaluation were prospective. Additionally, the same samples should ideally be evaluated on all assays, however volume restrictions precluded this direct comparison. As a result of these limitations, the idealized performance experienced in a laboratory setting may not be reproduced under field conditions. Another limitation of these results is that the performance of test devices can be influenced by several study specific variables: the reference methodology chosen, the type of samples collected, the PSO day, and the circulating serotypes and strains represented in a given evaluation panel. The Bio-Rad, SD, and to a lesser extent, Panbio NS1 tests have all been evaluated by multiple groups and our results are in broad agreement with previously published retrospective studies evaluating these test articles [48], [49]. To our knowledge, this is the first published report describing the performance of InBios NS1 assays. This is also the first time Panbio Rapid NS1 tests have been evaluated using circulating DENV samples from South America. The sensitivity for NS1 tests reported in the literature can vary based on study design and the reference method used, from 58–99% for RDTs and 37–93% for ELISAs. This complicates side-by-side comparison of our data with previously reported results. Our evaluation did reveal sensitivities higher than the median values reported which may be due to our use of virus isolation instead of qRT-PCR as the reference method. Because qRT-PCR can be more sensitive than virus isolation, our positive specimens may have possessed higher viremia, resulting in better overall sensitivity. Future work will need to focus on prospective evaluation of NS1 tests in clinical settings, located in varied geographic locations representing a broad variety of circulating DENV strains.

## Supporting Information

Table S1 **Raw data.**
(XLSX)
